# Fourier tools for the evaluation of refractive multifocal designs

**DOI:** 10.1038/s41598-023-50172-7

**Published:** 2023-12-19

**Authors:** Pablo De Gracia

**Affiliations:** School of Optometry, University of Detroit Mercy, Novi, 48377 MI USA

**Keywords:** Health care, Medical research, Optics and photonics

## Abstract

This paper presents innovative tools and methodologies for the theoretical assessment of optical properties in refractive multifocal designs. Utilizing lens segmentation techniques and classical Fourier optics, these tools can be of help evaluating multifocal contact lenses, intraocular lenses, small aperture designs, and corneal inlays. As an example of their utility, this study presents the through-focus Visual Strehl ratios in the frequency domain of 12 multifocal contact lenses from four companies, derived from the sagittal power profiles obtained with a NIMO equipment (LAMBDA-X) for three base prescriptions (− 6.00 D, − 3.00 D, and + 1.00 D). The contact lenses are also assessed alongside higher-order aberrations obtained from 65 eyes, measured using a Wavefront Sciences Complete Ophthalmic Analysis System (AMO). Diameter variations, corresponding to individual pupil sizes (2.45–6.27 mm), were considered in the evaluation. These novel tools enable the theoretical evaluation of multifocal solutions without the need for prototypes. In the case examples presented, they differentiate between lenses tailored for different presbyopic age groups, offer guidance on optimizing hyperfocal distance in contact lens design, and underscore the relevance of the effective aperture effect. Notably, this paper introduces the pioneering conversion of sagittal powers of multifocal solutions into an equivalent wavefront and optical quality metric, with potential applications in myopia control assessments. The author hopes that readers recognize and utilize these tools to advance the field of refractive multifocality.

## Introduction

Several instruments capable of obtaining optical profiles of multifocal solutions are currently available to researchers^1–5^. The sagittal power of the optical profiles of multifocal contact lenses is increasingly made public, making it possible to infer the optical properties of commercial designs. It is anticipated that in the upcoming years a similar trend will occur with IOLs^6^. The field of optical design has classically been interested in constructing optical quality metrics able to establish a connection between experimental measurements of Visual Acuity and optical characteristics of the human eye^7–9^. Zernike polynomials are often used in the assessment of the optical properties of the eye since they are a good match for the continuous wavefront of a healthy eye, and from them almost all the optical quality metrics of the eye can be constructed. This set of polynomial functions offers the advantage of accurately representing many of the most common eye refractive errors^10,11^. Second-order Zernike polynomials correctly represent myopia, hyperopia, and astigmatism. Third-order vertical and horizontal coma terms can be used to model misalignments occurring between cornea and crystalline lens, and fourth and sixth-order spherical aberration terms can account for asphericity values typically found in the optical structures of the eye.

Current multifocal refractive lenses use different areas of the pupil to provide clear vision at different distances, each of these areas potentially having different optical properties, such as radius of curvature^1,12–16^. Common terms used presently to refer to multifocal solutions include center-distance (CD) or center-near (CN), corresponding to designs in which the center and the surround areas of the pupil are matched with lens areas that have significantly different radius of curvature (aiming to provide clear vision either for distance or near). When trying to represent a multifocal wavefront for a CD or CN lens produced with Zernike coefficients, the fit is defective (i.e. in multifocal solutions two continuous zones can be discontinuous and present radically different sagittal powers that cannot be represented with a single set of Zernike polynomials). This is one of the major reasons for the lack of a procedure to derive an optical quality metric from optical profiles produced by optical metrology instruments such as NIMO^17^.

During recent years, our group has explored techniques to segment the eye entrance pupil that allow introducing different sets of Zernike polynomials to each of the areas^12–14^. This pupil segmentation allows using multiple sets of Zernike polynomials (one per subarea) with several levels of defocus to represent one entrance pupil. With this technique, discontinuities representing radically different sagittal powers, can be properly represented. Furthermore, a full customization of Zernike coefficients for each Zernike polynomial on each subarea can be performed independently.

**Figure 1:**
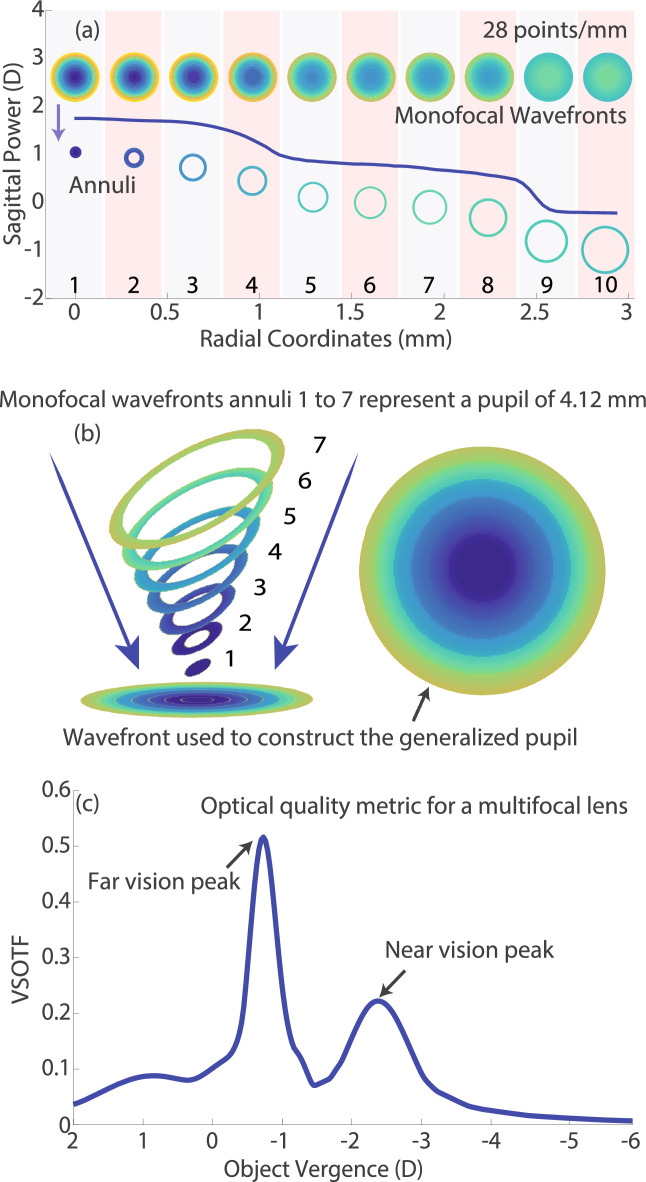
Visual representation of the pupil segmentation procedure followed to construct a multifocal optical profile. (**a**) Optical profile obtained from the NIMO instrument, with 28 points/mm that represent the sagittal power of the lens at each radial coordinate. Upper insets represent monofocal wavefronts, each corresponding to the average level of defocus obtained for ten representative locations (shaded bars) from the 28 points/mm measured, lower insets characterize the cropped annular areas. (**b**) Graphic representations of the construction and final product of a multifocal wavefront, (**c**) VSOTF obtained in the image plane for the optical profile shown in (**a**) and the multifocal wavefront shown in (**b**) with no added eye aberrations. The above graphs have been created for a pupil size of 4.12 mm (average pupil size for our subject database).

Figure 1 represents the basics of the procedure outlined in this paper. An optical profile is obtained from the NIMO, with a density of measurements of 28 points per millimeter across the diameter of the contact lens. This profile is a radial average of a sagittal (axial) power map, and it describes the intersection of light rays originating at any given point in the pupil with the optical axis. One wavefront is created per radial location measured in the optical profile and then a wavefront for a complete pupil is created with a single value of sagittal power, the one present at each radial location. For illustration purposes, in Fig. 1a only 10 radial locations (columns in grey and reddish backgrounds) are represented by their matching wavefront. The annuli that correspond to each of the radial values of sagittal power are cropped from the respective wavefront (lower line of insets in Fig. 1a) and then coupled with the rest of annuli (each representing the corresponding sagittal power for a particular pupil radius interval) to reconstruct a multifocal wavefront out of multiple monofocal wavefronts (Fig. 1b). It is important to note that spatial integration along the pupillary area of the annuli was performed, guaranteeing that spatial overlapping did not occur. Once a multifocal wavefront was created, a traditional Fourier optics procedure^18^ was followed to obtain the Visual Strehl in the frequency domain (VSOTF) (Fig. 1c)^19^. In essence, the VSOTF metric results from the multiplication of the Optical Transfer Function by a neural contrast sensitivity function.

When evaluating multifocal corrections, it is of interest to investigate them for a wide range of working distances, as can be done through this method by adding the particular vergence power corresponding for a given working distance as a base power to the multifocal wavefront. Through-focus evaluation has been performed in this paper for object vergence values ranging from + 2.00 D (working distance located 0.50 m beyond infinity) to -6.00 D (working distance located − 16.6 cm in front of the eye). Distances beyond infinity have been included with dual purpose: 1- to have a full evaluation of the whole through-focus curve (distances beyond infinity could come into play if the eye has a certain degree of residual accommodation), and 2- to give dioptric room to correct for the eye’s spherical equivalent prescription.

The market offers a rich variety of multifocal contact lenses, and it was reasonably expected that separate manufacturers would have produced particular solutions for aiding the presbyopic patient to optimize their optical performance through-focus (known as defocus curve in clinical practice)^15,16^. Also, it was reasonable to expect that presbyopes of varying ages and therefore with distinct levels of residual accommodation would need unique addition prescriptions. Therefore, the study lenses cover four of the main manufacturers of contact lenses worldwide, and two-to-four models each of them manufactures for patients ranging from early presbyopes to patients who are > 65 years old within the same product line. This is the list of lenses in the study: two Bausch & Lomb lenses (Ultra high and Ultra Low), three Alcon lenses (Air Optix Aqua high, Air Optix Aqua medium, Air Optix Aqua low), three Johnson and Johnson lenses (Oasys high, Oasys Medium, Oasys Low), and four CooperVision lenses (Biofinity 2.50 N, Biofinity 2.00 N, Biofinity 1.50 N & Biofinity 1.00 N).

Potential variability across prescription powers has also been investigated in this study. It is expected that a given model of contact lens will preserve a fixed multifocal profile across all refractive prescription levels. However, a − 3.00 D myope needs a lens with different radius of curvature and central thickness than a + 6.00 D hyperope. Therefore, changes in far contact lens prescription power could potentially produce changes in the multifocal profile. Three different far prescriptions (− 6.00 D, − 3.00 D, and + 1.00 D) have been evaluated with the NIMO instrument.

These 12 multifocal designs were evaluated in combination with the higher-order aberrations (HOAs) of a population of 65 eyes. By including 65 eyes we accounted for inter-subject variability and provided a reasonably solid average contact lens performance that allows for a fair comparison between lenses. Measurements of HOAs also have inherent intra-subject variability but since intra-subject variability is normally one order of magnitude lower than inter-subject, having 65 eyes on this study protects from artifacts arising from this unique source of error.

In summary, this paper presents the mathematical details for the implementation of the required pupil segmentation techniques and classical Fourier optics required to evaluate multifocal corrections. Also, a demonstration of the potential applications of this method is presented, through the evaluation of 12 commercial multifocal contact lenses, with three different far prescriptions (− 6.00 D, − 3.00 D, and + 1.00 D), and in combination with the HOAs of 65 eyes. All simulations presented include a total of 36 multifocal scenarios with 2340 individual subject cases.

## Methods

### Measurements of the optical profile of contact lenses

The NIMO equipment was used to measure the optical profiles of the 12 multifocal lenses in three different base corrections (− 6.00 D, − 3.00 D, and + 1.00 D as labelled by the manufacturer). Measurements with NIMO are based on the Phase-Shifting Schlieren technique. This technique combines the Schlieren principle with the phase-shifting technique that is used in interferometry by using a Schlieren filter and an adapted set-up. After the application of the phase shift technique, the Schlieren phase was calculated and converted into beam deviation values^17^.

**Figure 2:**
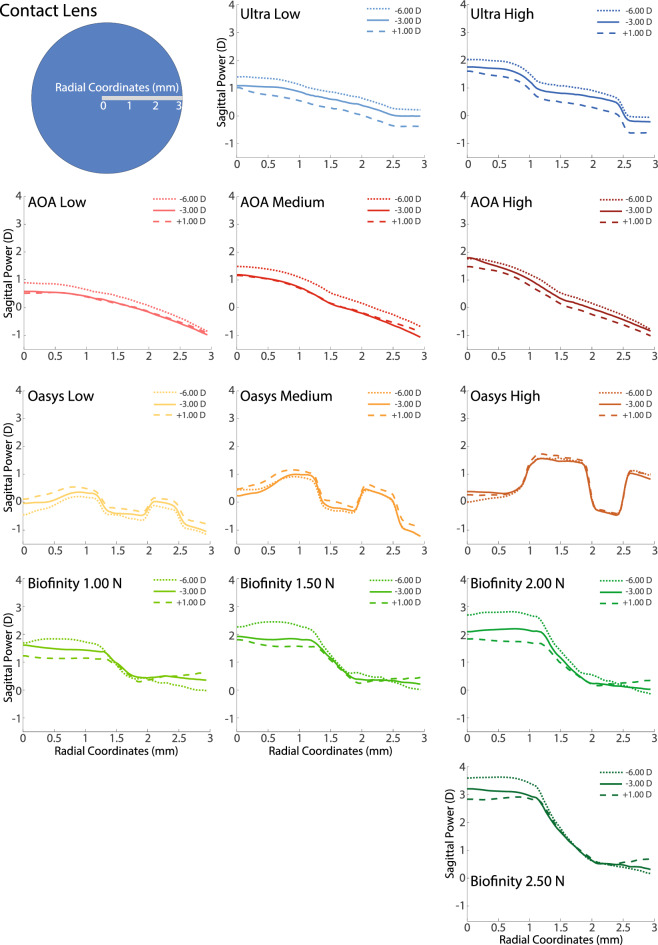
The sagittal power of the 12 lenses of the study for the three base corrections measured—− 6.00 D (dotted line), − 3.00 D (solid line), + 1.00 D (dashed line)—as a function of the radial coordinates. Base corrections have been removed from the power profiles shown. The columns from left to right show increasing levels of addition that are available for each family of lenses. The blue circle represents a contact lens where the radial coordinates of NIMO measurements are represented in grey with 0 mm being placed at the center of the lens.

The sagittal powers as a function of the radial coordinate of the 12 multifocal lenses of the study are represented in Fig. 2: two Bausch & Lomb lenses (Ultra high and Ultra Low), three Alcon lenses (Air Optix Aqua high, Air Optix Aqua medium, Air Optix Aqua low), three Johnson and Johnson lenses (Oasys high, Oasys Medium, Oasys Low), and four CooperVision lenses (Biofinity 2.50 N, Biofinity 2.00 N, Biofinity 1.50 N & Biofinity 1.00 N).

### Eye population description

**Figure 3:**
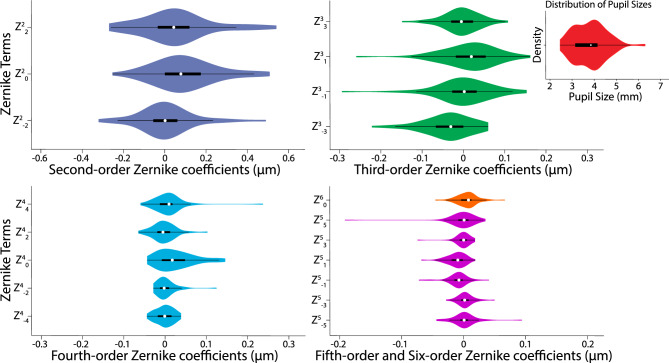
Low and high order aberrations of the population of 65 eyes in our study. They are presented following OSA convention^23^. Upper left panel shows the distribution of Zernike coefficients for second-order optical aberrations. Upper right panel presents third-order coefficients. Bottom left panel displays fourth-order coefficients. Bottom right panel shows fifth-order coefficients in pink and sixth-order spherical aberration in orange. Inset in the upper right corner (red) represents the pupil sizes of the population of the study. White circles show the medians, thin bars extend to 5th and 95th percentiles, wide bars indicate the 25th and 75th percentiles and the polygons represent density estimates of data and extend to extreme Zernike coefficient or extreme pupil size values.

A database with the optical aberrations of 65 healthy eyes provided by Bausch & Lomb was used in this study. The participants in our study span an age range of 49–63 years, with a mean age of 52.6 years. This eye database was collected by using a Wavefront Sciences Complete Ophthalmic Analysis System (AMO), this procedure is non-invasive and only requires the light of a super luminescent diode to enter and exit the eye^10^. The procedure to collect the information on these patients adhered to the tenets of the Declaration of Helsinki and to all federal and state laws applicable in New York State. Aberrations included second, third, fourth, and fifth-order aberrations plus sixth-order spherical aberration (also known as secondary spherical aberration). The importance of HOAs in vision diminishes with their order. In this paper, secondary spherical aberration was included in the simulations for its known interactions with other radially-symmetrical aberrations such as defocus and fourth-order spherical aberration^20,21^, which could be of particular interest when elucidating the multifocal properties of ophthalmic devices. Figure 3 contains the description of the aberrations of the 65 eyes of this study^22^.

For the simulations in this paper, the eye database had their equivalent sphere corrected. This was achieved by shifting the maximum of the VSOTF obtained for each eye with the monofocal lens to 0.00 D vergence (distance correction). Subsequently, the determined shift for the monofocal lens was applied uniformly to the 12 multifocal lenses. The amount of shift was particular to each eye but constant for all the lenses on the same eye.

### Pupil segmentation techniques

The mathematical expressions that allow segmenting the eye’s pupil radially and angularly have been presented elsewhere^12,13^. In short, a multifocal wavefront with radial symmetry can be generated by creating monofocal wavefronts with different defocus values, cropping the areas of interest for each of the corresponding defocus values, and adding spatially all the cropped annuli. Equations 1 and 2 show the mathematical transcription of the procedure:

1 $${W}_{CL}=\sum_{j=1}^{n}{w}_{j}\left({D}_{j}\right)* {\sigma }_{j} \left(l\right)$$

2 $${\sigma }_{j}\left(l\right)=\left\{\begin{array}{l}\begin{array}{l} 1,\quad \forall \rho \in \left[\frac{Rl}{n},\frac{R\left(l+1\right)}{n}\right]\end{array}\\ 0,\quad otherwise\end{array}\right.$$

where ${W}_{CL}$ represents a multifocal contact lens wavefront that is compounded by adding monofocal wavefronts ${w}_{j}$ with a defocus value (${D}_{j}$) obtained from the sagittal powers measured by the NIMO and $j$ denotes the radial coordinate. From each of these monofocal wavefronts, the appropriate radial areas are selected by multiplying ${w}_{j}$ by a masking function ${\sigma }_{j}$ that has a value of one for a particular annulus and takes the value of zero otherwise. Where $R$ represents the total radius measured of the lens and $\rho$ is the radial coordinate.

Where $l$ is linked to the number of radial zones and covers all integer values from (0 to $n-1$). $n$ is the number of radial areas used to represent the optical profile that is equal to the number of points measured by the NIMO in the multifocal lens and always assumed to be greater or equal than 2. Note that, within the same lens a particular $\rho$ and $l$ will have a uniquely defined ${D}_{j}$ obtained from the NIMO measurement. However, when changing from one lens to another the same combination of $\rho$ and $l$ will lead to a different value ${D}_{j}$. Also, to note, $n$ = 1 would represent a monofocal lens and a simple ${W}_{CL}={w}_{1}\left({D}_{1}\right)$.

The multifocal wavefront ${W}_{CL}$ was introduced in the expression of the generalized pupil from which different optical quality metrics can be calculated^18^.

### Metrics used to compare the study lenses

In this paper we have evaluated 12 multifocal and one monofocal designs by using the metric called Visual Strehl Ratio in the frequency domain as described in Eq. 3^7,19^.

3 $$VSOTF=\frac{{\int }_{0}^{2\pi }{\int }_{0}^{60}CSF*\left|Re\left(OTF\right)\right| dw d\theta }{{\int }_{0}^{2\pi }{\int }_{0}^{60}CSF*{OTF}_{DL}dw d\theta }$$

where CSF stands for contrast sensitivity function, OTF represents optical transfer function, θ characterizes angles, and $w$ represents frequencies. Integration occurs for all angles (0–2 $\pi$) and frequencies between 0 and 60 (VA = 20/10) cycles per degree. The contrast sensitivity function used for the calculations in this paper accounts only for the neural part of visual processing and was obtained from interferometric measurements available in the scientific literature^24,25^. The matrix size used in the simulations was 512 × 512 pixels.

Equation 4 shows the description of the procedure when eye aberrations are included in the simulations.

4 $${W}_{T}={W}_{CL} +{W}_{Eye}$$

where ${W}_{T}$ represents a wavefront superposition in the pupil plane of the multifocal profile ${(W}_{CL})$ of the contact lens and the eye’s aberrations ${(W}_{Eye})$. This was achieved by using ${w}_{j(CL+Eye)}$ in Eq. 1 that contained both the sagittal power of the multifocal contact lens measured by the NIMO and the optical aberrations for each of the 65 eyes. From each of these wavefronts, the appropriate annuli are selected by multiplying ${w}_{j(CL+Eye)}$ by a masking function ${\sigma }_{j}$, equivalent to the one defined in Eq. 2. ${\sigma }_{j}$ has a value of 1 for a particular annulus and takes the value of zero otherwise. Where *R* represents the maximum radius of the calculation (the smaller of the pair: pupil radius, contact lens radius measured by the NIMO). Where n, $\rho$,and l represent the same values as in Eq. 2.

The multifocal wavefront ${W}_{T}$ loaded with the eye database HOAs was then introduced in the expression of the generalized pupil from which the VSOTF shown in Eq. 3 was calculated. The combination of eye plus the multifocal design has been considered as a single VSOTF because the image created by the multifocal correction keeps the phase information intact (i.e. no intermediate image is created on a screen) when interacting with the optics of the eye.

To establish comparisons between lenses, three metrics have been defined: area under the through-focus VSOTF, range above threshold, and peak performance. The definition of these three metrics can be seen in Eqs. 5, 6, 7, and 8.

5 $$Area under {VSOTF}_{TF}=\sum_{-1}^{5}VSOTF$$

6 $$Range above threshold= \sum_{-1}^{5}{x}_{d}$$

7 $${x}_{d}\left(d\right)=\left\{\begin{array}{l}\begin{array}{c} \frac{6}{193} D, \quad \forall VSOTF\left(d\right)>0.12\end{array}\\ 0, \,\,\,\,\,\,\,\,\,\,\,\,\,\quad \forall VSOTF\left(d\right)<0.12\end{array}\right.$$

where 6/193 diopters for ${x}_{d}$ comes from the number of samples used to describe the through-focus curves (6.00 D of interval and 193 sample points). With 193 samples we have used a defocus step through-focus of 0.0313 D and guaranteed that simulations test the 0 vergence value. Selecting 32 samples per diopter achieved a favorable balance between precision and calculation times. A VSOTF threshold of 0.12 predicts a level of logMAR visual acuity of 0.20^26^, and it has been used abundantly in the literature^12,26,27^. Analogous calculations for other logMAR levels could be performed by varying the level of the threshold of the VSOTF. Values under 0.12 are not considered for the interval of acceptable vision but are still of some utility (corresponding logMAR values between 0.20 and 0.40, 6/9 to 6/15 or 20/30 to 20/50 Snellen)^26^ and therefore counted on the area under the curve metric.

8 $$Peak Performance={\text{max}} \left(Throughfocus VSOTF\right)$$

In summary, through-focus (-1.00 D to + 5.00 D) monocular VSOTFs (Eq. 3) were calculated for the 12 multifocal commercial designs described in Fig. 2 in combination with the 65 eyes described in Fig. 3 and with one monofocal design that serves as a reference case.

## Results

**Figure 4:**
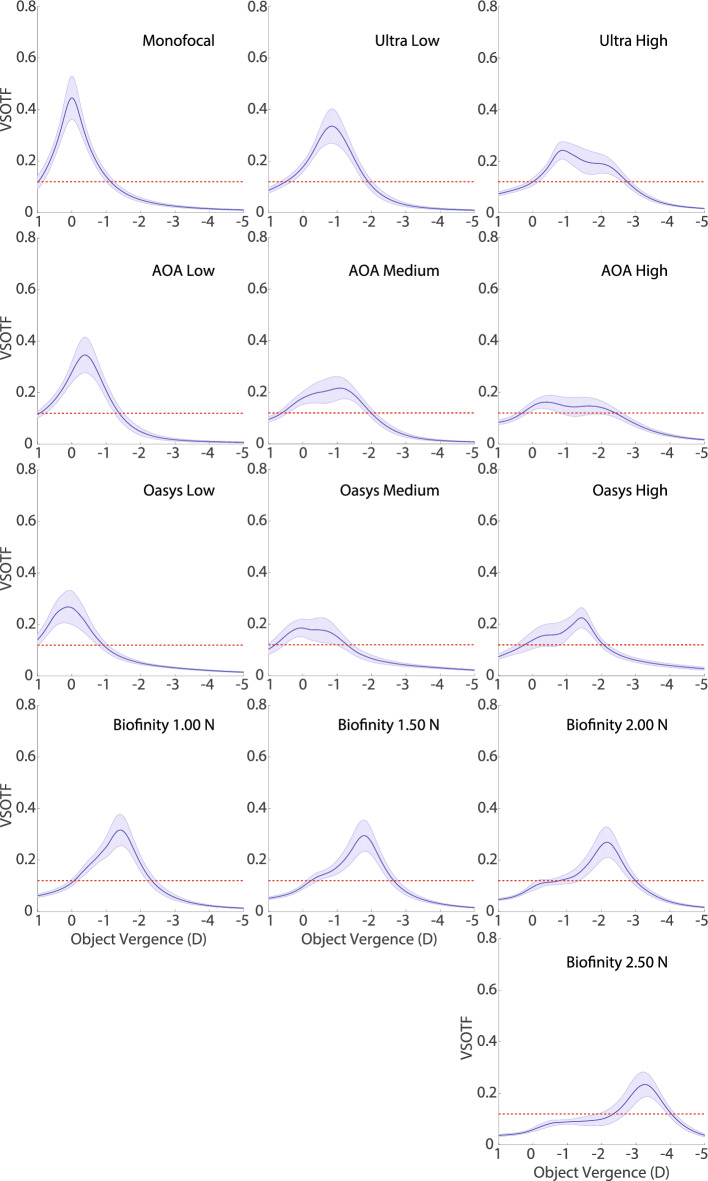
Average through-focus VSOTF metric for the lenses under study when combined with the data set of eye aberrations. The range through-focus range includes object vergences between 1.00 and − 5.00 D. Shaded areas represent ± ½ of the standard deviation for each lens across the 65 eyes. Red dashed lines represent a 0.12 threshold for acceptable vision in the VSOTF metric that roughly corresponds to a visual acuity of 20/32.

Consequently, we calculated a total of 2340 monocular multifocal through-focus VSOTF curves (comprising 12 multifocal designs across 65 subjects with 3 base corrections each). Furthermore, 65 monocular monofocal through-focus VSOTF curves were computed for comparative analysis (1 design across 65 subjects).Fig. 4 shows the average performance through-focus of each of the lenses, with the shaded areas representing ± ½ of the standard deviation for each lens across the 65 eyes. We want to focus our interest on the multifocal nature of the profile more than in the refractive correction of the lens, therefore the nominal − 6.00 D, − 3.00 D and + 1.00 D, base corrections for distance have been removed previously to performing the calculations.

Upon direct visual inspection of Fig. 2 the reader can observe that no systematic remarkable differences are observed between distance prescription powers, with the potential exception of the − 6.00 D prescription offering more sagittal power than the − 3.00 D (0–0.50 D), and the + 1.00 D prescription offering less sagittal power than the − 3.00 D (0.05–0.40 D). This difference roughly translates into a shift of the overall VSOTF curves from one distance prescription to another. Therefore, in Fig. 4 only the through-focus VSOTFs results obtained for the − 3.00 D distance correction are presented. Data obtained for the − 6.00 D and + 1.00 D prescriptions will be made available to sensible direct requests to the author of this study.

**Figure 5:**
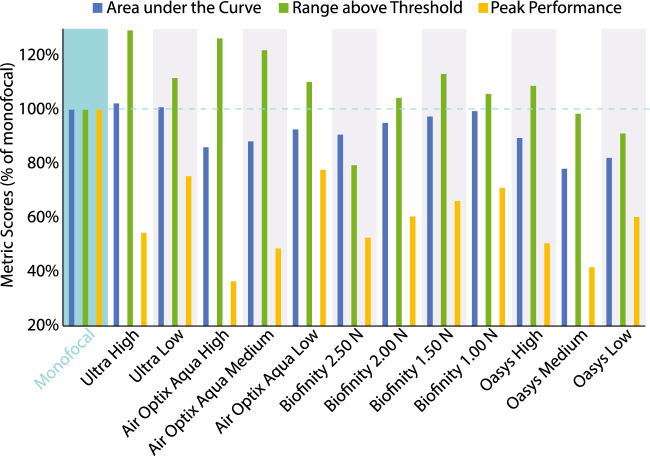
Average performance of 13 lenses (one monofocal and 12 multifocal) for our population of 65 eyes. This graph shows area under the VSOTF through-focus, range above threshold, and peak performance as defined by Eqs. 5, 6, and 8 respectively. All of them normalized by the corresponding value obtained for the monofocal lens. Dashed light blue line represents 100%; values larger than 100% indicate a larger value than the one obtained with the monofocal lens. Shaded blue indicates monofocal values, alternating gray and white shading is intended to help with visualization.

Figure 5 shows the average performance of the 12 lenses of the study and a control case (12 multifocal and one monofocal) for our population of 65 eyes. This figure shows three metrics as defined by Eqs. 5, 6, and 8: area under the VSOTF through-focus, range above threshold, and peak performance.

Nine out of the 12 multifocal designs offer a larger range above threshold than the monofocal design: ​Ultra High (129%), Ultra Low (112%), Air Optix Aqua High (126%), Air Optix Aqua Medium (122%), Air Optix Aqua Low (110%), Biofinity 2.00 N (104%), Biofinity 1.50 N (113%), Biofinity 1.00 N (106%), and Oasys High (109%). Three out of the 12 designs offer an area under the curve within a 2% of the one offered by a monofocal lens: Ultra High (102%), Ultra Low (101%), Biofinity 1.00 N (99.4%). All lenses offer a peak performance that is lower than the one provided by a monofocal lens, with the three best performers in this aspect being Air Optix Aqua Low (78%), Ultra Low (75%), and Biofinity 1.00 N (71%).

**Figure 6:**
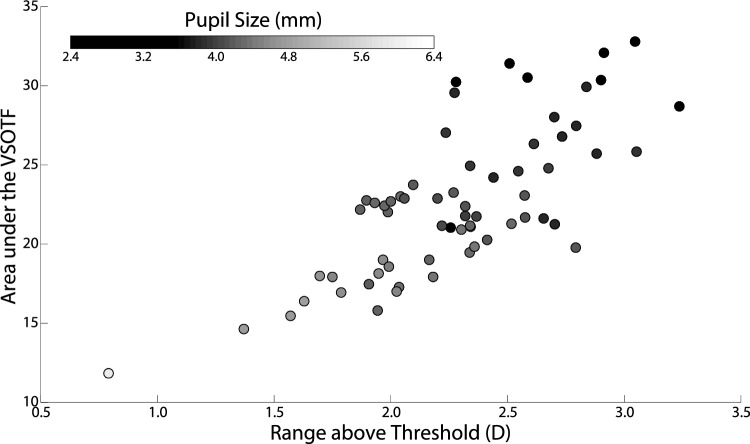
Area under VSOTF as a function of the range above threshold of each of the 65 eyes averaged for the 12 multifocal designs of the study also as a function of their pupil size (gray scale bar).

The optical performance as a function of the area under the VSOTF and the range above threshold of each of the 65 eyes of our study population averaged across the 12 multifocal lenses in reference to the eye’s pupil size (gray scale bar) is shown in Fig. 6.

## Discussion

### Lens characteristics

This paper clearly shows the existing tradeoff between peak performance and range above threshold through-focus provided by a particular lens. Early presbyopes can use residual accommodation to move the curves provided by multifocal corrections, therefore designs targeted to early presbyopes (Ultra Low, Air Optix Aqua low, Oasys Low, Biofinity 1.50 N & Biofinity 1.00 N), exhibit a higher peak performance that can be used in combination with residual accommodation to overcome the generally shorter interval through-focus above threshold that these lenses offer. On the contrary, lenses that are created for older presbyopes, whom have no residual accommodation left, offer larger intervals of optical quality above threshold, and sacrifice peak performance (Ultra high, Air Optix Aqua high, Oasys high, and Biofinity 2.50 N). Also, an intermediate version exists for presbyopes with some but not a large amount of residual accommodation (Air Optix Aqua medium, Oasys Medium, Biofinity 1.50 N & Biofinity 2.00 N).

The peak performance of all lenses in the study, with the exceptions of the Oasys Low and the Oasys Medium models, occurs at a distance from the patient between 80 cm and 2 m (vergences of 1.25 D and 0.50 D respectively). Placing peak performance closer than infinity allows for better use of the interval of good quality of vision occurring on both sides of the work distance at which peak performance occurs (optimization of the concept of hyperfocal distance).

Of special interest are the Biofinity “N” presented in this study: their through-focus curves explain why they are to be used in combination with the “D” versions. The “N” lenses seem to disregard the optical quality obtained for distance correction, and focused on providing acceptable quality of vision at near; they are CN designs. Biofinity “D” lenses designed to be used in the dominant eye carry the distance correction on the center of the lens (CD); this is the opposite of the Biofinity lenses for the non-dominant eye “N” presented on this study. “D” lenses will provide the optical quality necessary for far vision. Together the “N” (CN) and “D” (CD) versions of the Biofinity lenses provide a modified monovision correction instead of a truly multifocal correction where both eyes will experience the same optical correction. Because of this monovision feature, and not having presented the characteristics of the “D” lenses in this manuscript, a direct comparison is difficult to be made with the rest of the study lenses and therefore the following discussion does not include them.

Oasys lenses show the importance of the effective aperture effect^28^. This effect basically predicts that the higher the level of change on the sagittal power across the radial coordinates in the entrance pupil, the lower the overall optical quality provided. Peak performance for Oasys Low lenses is 21% lower than the one offered by Ultra-Low and 23% lower than the one offered by Air Optix Aqua Low lenses. Interval through-focus above threshold for Oasys Low lenses is 18% lower than the one offered by Ultra-Low and 17% lower than the one offered by Air Optix Aqua Low lenses. Area under the curve for Oasys Low lenses is 19% lower than the one offered by Ultra-Low and 11% lower than the one offered by Air Optix Aqua Low lenses. Although the sagittal power shown in the third row of Fig. 2 shows two foci for the Oasys Lenses, there are three main reasons why their optical performance will not clearly display the expected two foci of clear vision. 1- The higher-order aberrations of the 65 eyes smooth the VSOTF of the lens alone, 2- As can be seen in Fig. 3, pupil sizes vary greatly between the 65 eyes, with a majority of our subject database having pupils under 4.25 mm, therefore the second increase of power in the profile is often not used, and 3- multiple transition areas between near and far powers contribute to smooth out the potential two foci design.

A clear trend appears between a smaller pupil size and a better performance through-focus on average across the 12 designs of the study. When dealing with a monofocal lens, smaller pupil sizes (up to 2 mm in diameter) reduce the amount of aberrations present in the eye and increase overall visual performance through-focus, for as long as diffraction does not become a dominant factor^29^. Figure 6 confirms that this effect is still present when multifocal lenses are used. Relative changes in performance between lenses in Fig. 5 are smaller in magnitude than relative changes in performance between pupil sizes in Fig. 6. This reinforces the predominant clinical practice whereby presbyopes are instructed by clinicians to maintain high levels of illumination when performing near tasks, and with natural evolution where senile myosis contributes to extend the depth of focus of elderly patients^30,31^. This phenomenon has allowed for the eruption of multiple technologies and pharmacological treatments in recent years that limit pupil size in order to increase a patient’s visual performance through-focus^32–36^.

In our study, we did not categorize lens performance based on specific pupil sizes due to the dynamic nature of pupils throughout a typical day, influenced mainly by varying luminance levels. Our rationale for focusing on average performance across a population of 65 subjects with diverse pupil sizes stems from the understanding that individuals naturally encounter both larger and smaller pupils in their daily activities. This variability, driven by changes in luminance, is a fundamental aspect of real-world conditions. Additionally, different pupil sizes exhibit distinct sets of higher-order aberrations, preventing specific interactions of a given wavefront from unduly influencing the overall study. However, we recognize the potential for full multifocal contact lens customization tailored to individual subjects based on ergonomics. Future studies could explore measuring a patient's pupil size as a function of luminance, considering factors such as daily hours of near work and characteristics of their typical environments. Such an approach could involve weighting the relative importance of specific pupil sizes, introducing the subject's unique wavefront, and either selecting the most suitable lens from the existing commercial designs or even generating an optimal multifocal lens for the individual.

### Utility of the proposed method

There have been attempts in the past to evaluate depth of focus of healthy subjects from wavefront measurements^37^. However, these early attempts did not include multifocal corrections. As explained above, wavefront reconstruction with a multifocal correction would not be properly fitted with Zernike polynomials unless the process presented in this paper is used. The method we are describing arises from some of our work published during the last decade^12,14,38^. However, this is the first time this method of converting sagittal power into the corresponding annuli of a multifocal wavefront (created with standard Zernike polynomials) is fully published in a paper format. The method allows the systematical evaluation of the optical performance of multifocal corrections through simulations, which do not require the implementation of optical benches. The central realization that makes this work valuable is the fact that sagittal power obtained with metrology devices can be represented locally with the corresponding wavefront obtained from Zernike polynomials. This was never presented in any of our previous papers^12,14,38^, and was not present in classical work^7,8,39^.

An approach to simulate multifocal corrections called temporal multiplexing has been recently introduced. Temporal multiplexing consists in presenting different refractive powers to the eye each of them ranging from a tenth of a microsecond to several microseconds^40^. Due to the short exposure time, each of the individual defocus values blends with others, the final integrated signal containing multiple defocus values. Temporal multiplexing does represent accurately the percentage of light carried by each refractive power. However, it uses the whole pupil for each refractive power represented and it is not capable of segmenting the pupil in different areas or creating any kind of asymmetries (neither radial nor angular). On the other hand, the method presented in this paper has the ability to represent any local distributions of power around the entrance pupil.

Other approaches to evaluate multifocal corrections were presented by Alarcon et al^41^., Vega et al^42^., Armengol et al^43^., Azor et al^44^., and Torres-Sepulveda et al^45^. In their works, the optical quality of intraocular lenses was evaluated by creating an optical system with an eye model. All these procedures do allow for testing multifocal designs but need a physical lens to be employed and experimental measurements to be taken in an optical bench. Furthermore, a recent study has shown an instrument capable of studying the optical capabilities of theoretical designs for spectacle lenses by using a spatial light modulator^46^. Although this type of instrumentation can reproduce multifocal patterns, it requires an initial investment, and will have difficulties when evaluating smaller optical areas, such as the ones needed to represent contact lenses. On the other hand, our method represents the optics of contact lenses, and intraocular lenses already present in the market and those that do not yet exist. To reproduce lenses that do not exist yet, we could substitute the sagittal powers of the 12 lenses used as input on this article by any other combination of sagittal powers.

Bakaraju et al^47^. have presented a method where by coupling a custom-made script in MATLAB with Zemax, the optical profiles obtained in a NIMO instrument can be evaluated. This is done through creating a super conic surface in the front surface of the correcting contact lens in their Zemax model. Their method requires the utilization of Zemax to be completed, which introduces extra steps and reduces control over the process. It is not clear from their publication if their method allows for inclusion of any given set of aberrations coming from a particular subject or whether they could have as output any desired optical quality metric^7^.

Although radial asymmetries are not present on the set of multifocal contact lenses reported in this paper, in the simplest case of radial asymmetries, multifocal lenses with a correction for astigmatism could be studied. The most straightforward way to deal with the presence of astigmatism is to model the individual wavefronts of Fig. 1b, with a certain level of astigmatism already imbued in them that could easily be obtained from NIMO readings. Ultimately, the limits for representing asymmetries in a lens with this method are: 1- the precision with which the NIMO (or any other) equipment can measure sagittal powers, and 2—the real-life utility of these constructions. Segmentation of the pupil could be done for any kind of sub-area considered of interest, by plugging a certain level of sagittal power, creating a wavefront representing it, and reconstructing the multifocal wavefront pixel by pixel if required. In short, the method proposed in this article is more cost effective, more flexible, and has more potential applications than the existing methods in the literature.

## Conclusion and future work

The algorithms introduced in this study serve as a crucial framework for assessing refractive multifocal designs through the integration of Fourier optics and Zernike-based wavefronts. The implications of this research extend beyond this paper current scope and can be applied to address challenges in myopia progression control. This includes evaluating the central and peripheral optical quality offered by multifocal contact lenses designed explicitly for myopia control, such as MYLO and MiSight. Furthermore, these algorithms can be adapted for exploring solutions involving small apertures, such as the IC-8 Intraocular Lens, Kamra Inlay, or pharmaceutical approaches aiming to reduce pupil size.

## Data Availability

The data and algorithms utilized in this study are not publicly accessible. The author will evaluate requests for information sharing on a case-by-case basis, considering reasonable inquiries. For those interested, the author's personal email is pablodegracia@gmail.com.
